# Experiences of Saudi Adults Living with Heart Failure: A Reflexive Thematic Analysis of Health-Related Quality of Life

**DOI:** 10.3390/healthcare14162458

**Published:** 2026-08-09

**Authors:** Nader E. Alotaibi, Omar Qaladi, Monirah Albloushi, Mahaman Laouali Moussa, Raied Alotaibi, Ahmed M. Al-Wathinani

**Affiliations:** 1 Department of Medical-Surgical Nursing, College of Nursing, King Saud University, Riyadh 12372, Saudi Arabia; 2 Department of Community and Psychiatric Mental Health Nursing, College of Nursing, King Saud University, Riyadh 12372, Saudi Arabia; 3 Department of Basic Science, Prince Sultan Bin Abdulaziz College for Emergency Medical Services, King Saud University, Riyadh 12372, Saudi Arabia

**Keywords:** heart failure, health-related quality of life, qualitative research, Saudi Arabia, patient experiences, spirituality, family support, chronic illness

## Abstract

Background: Heart failure is a chronic and progressive condition that substantially affects patients’ physical, emotional, social, and spiritual well-being. Although the clinical burden of heart failure is well documented, limited qualitative evidence exists regarding how Saudi adults experience and interpret the condition within their cultural, familial, and religious contexts. This study explored the experiences of Saudi adults living with heart failure and their perceptions of its impact on health-related quality of life. Methods: A qualitative descriptive study informed by an interpretivist perspective was conducted using semi-structured interviews with 25 Saudi adults diagnosed with heart failure. Participants were recruited from specialized heart failure and cardiac outpatient clinics within a tertiary medical city in Riyadh, Saudi Arabia. Interviews were audio-recorded, transcribed verbatim, and analyzed using Braun and Clarke’s reflexive thematic analysis. Results: Four themes were identified: (1) emotional distress and fear, (2) loss of independence and increased dependency, (3) social role disruption, and (4) coping and adaptation strategies. Participants described significant psychological burden, reduced autonomy, and changes in family and social roles following their diagnosis. Conclusions: Heart failure was experienced as more than a physical illness, affecting participants’ emotional well-being, social identity, family relationships, and spiritual life. The findings underscore the importance of holistic and culturally responsive care that addresses psychological, social, spiritual, and family-related needs alongside symptom management.

## 1. Introduction

Heart failure (HF) is a chronic, progressive, life-limiting illness affecting millions of adults throughout the world and representing one of the leading causes of hospitalization, disability, and premature mortality [1,2]. Despite significant advances in the pharmacological and technological management of HF, it remains a profoundly disruptive illness—one that reshapes bodies, routines, identities, and overall well-being [3,4]. For many, HF not only weakens the heart but also changes the rhythms of everyday living: mobility is constrained, social roles are altered, and emotional and existential uncertainty is generated [5,6]. Such burdens are often intensified in cultural contexts where family responsibilities, religious beliefs, and social expectations play a central role in shaping experiences of illness [7,8].

The prevalence of HF continues to increase globally, with approximately 56 million people currently living with the condition worldwide [9,10]. In the general adult population, estimates of prevalence usually range from 1% to 3% [9,10,11]. In the Arab and Middle East region, there is greater variation in prevalence. For example, in the region, the prevalence of HF in 2019 was estimated at approximately 0.78% in Saudi Arabia, whilst in other countries it was slightly higher, such as 1.00% in Kuwait [12]. In the case of Saudi Arabia, the prevalence of chronic HF in the population is approximately 1.61% based on available evidence [13]. Earlier estimates suggested that HF affected approximately 1.2% of the Saudi population, equivalent to around 320,000 individuals at that time [13]. These statistics are, in all likelihood, an ever-increasing burden because of the rising prevalence of HF risk factors such as hypertension, diabetes, and obesity [14,15]. Although clinical and epidemiological data provide important insight into the burden, prevalence, and outcomes of HF, they offer only a partial understanding of how individuals experience and live with the condition. The lived experience of HF, particularly its impact on daily life, emotional well-being, social functioning, and Health-Related Quality of Life (HRQoL), remains underrepresented in the literature.

Although a growing body of qualitative research has examined the lived experience of heart failure, much of this work has been conducted in Western contexts, where healthcare systems, cultural norms, and social structures differ substantially from those in the Middle East [6,16]. Existing qualitative studies consistently describe heart failure as a multidimensional experience characterized by physical limitation, emotional distress, and social disruption, including reduced participation, uncertainty about the future, and altered daily functioning [6]. Moreover, qualitative syntheses and phenomenological studies have highlighted how heart failure profoundly affects identity, autonomy, and patients’ perceptions of normality, often framing the condition as a life-altering and existential experience.

However, despite these important insights, the existing literature provides limited understanding of how such experiences are shaped within culturally specific contexts. In particular, prior research has insufficiently examined how sociocultural factors such as family interdependence, religious beliefs, and socially defined roles influence patients’ interpretations of illness and their HRQoL. This limitation is notable given evidence that heart failure significantly disrupts social roles and participation, often leading to dependency, isolation, and changes in family dynamics [17,18]. Yet, the cultural meanings attached to these changes, especially in non-Western societies, remain underexplored.

In Middle Eastern settings, and particularly in Saudi Arabia, illness experiences are embedded within a sociocultural framework characterized by strong family cohesion, religious centrality, and collective identity [19]. These contextual factors may fundamentally shape how individuals interpret dependency, negotiate social roles, and engage in coping processes. Nevertheless, qualitative research examining heart failure within this context remains limited and lacks in-depth interpretive exploration of the intersection between culture, spirituality, and lived experience [20]. Consequently, it remains unclear whether existing conceptualizations of heart failure adequately capture the culturally embedded dimensions of HRQoL in this population.

Addressing this gap, the present study extends beyond a geographically specific replication of prior findings by explicitly examining how Saudi adults interpret and experience heart failure within their cultural, familial, and religious contexts. By employing Braun and Clarke’s reflexive thematic analysis, this study aims to generate contextually grounded insights into how HRQoL is constructed through the interplay of physical, social, emotional, and spiritual dimensions.

## 2. Methods

### 2.1. Study Design

This study adopted a qualitative descriptive design informed by an interpretivist perspective to explore how Saudi adults experience and make sense of living with HF and its impact on HRQoL. A qualitative descriptive approach was considered appropriate because it enables the generation of rich, practice-relevant accounts of participants’ experiences while remaining grounded in their own perspectives and everyday realities [21,22].

The study was underpinned by this interpretivist orientation, which acknowledges that experiences of illness are shaped by personal, cultural, social, and religious contexts. Consistent with this epistemological position, Braun and Clarke’s reflexive thematic analysis (RTA) was selected because it conceptualizes themes as interpretive patterns of shared meaning that are actively developed through researchers’ reflexive engagement with the data, rather than as objective categories waiting to be discovered [23,24]. This approach was particularly appropriate for exploring how participants constructed and interpreted the personal, familial, cultural, and spiritual meanings of living with heart failure while explicitly recognizing the researchers’ active role in knowledge construction throughout the analytic process [25]. This study is reported in accordance with the Consolidated Criteria for Reporting Qualitative Research (COREQ) guideline [26].

This study was grounded in an interpretivist epistemological perspective, which posits that reality is not objective but is constructed through social interaction and shaped by cultural, contextual, and experiential meanings. Within this epistemological perspective, a qualitative descriptive approach was employed to generate a detailed, practice-relevant account of participants’ experiences, closely reflecting their everyday language and viewpoints. The analysis was informed by Braun and Clarke’s reflexive thematic analysis (RTA), which provided an interpretive framework for constructing themes as patterns of shared meaning through researchers’ active and reflexive engagement with participants’ accounts. This methodological integration is coherent because qualitative description accommodates varying levels of interpretation while remaining closely grounded in participants’ accounts. In this study, RTA facilitated the identification and development of themes that extended beyond descriptive accounts, enabling deeper insight into patterns of shared meaning, particularly regarding the cultural, familial, and spiritual dimensions of living with heart failure in the Saudi Arabian context.

### 2.2. Participants

A purposive sampling strategy was used to recruit Saudi adults diagnosed with HF who could provide rich and diverse perspectives regarding the impact of the condition on their daily lives and HRQoL. Participants were eligible if they were aged 25 years or older, had a confirmed diagnosis of HF with either reduced or preserved ejection fraction, were clinically stable at the time of recruitment, and were able to communicate in Arabic or English. Participants with severe cognitive impairment, active psychiatric illness, advanced malignancy, or other critical comorbid conditions that could substantially influence their experiences independently of HF were excluded.

Maximum-variation sampling was employed to capture a broad range of experiences across demographic and clinical characteristics, thereby enhancing the transferability of the findings. Participants were recruited to reflect variation in age, gender, educational background, employment status, duration of illness, and geographical background (urban and rural settings), enabling the inclusion of diverse perspectives on living with HF within the Saudi sociocultural context.

Sample size was guided by the principle of information power, whereby the adequacy of the sample was determined by the study aim, sample specificity, quality of dialogue, and richness of the data generated [27]. Sufficient information power was evaluated through ongoing assessment of the depth, richness, and relevance of interview data during concurrent data collection and analysis. The research team considered the specificity of the sample, quality of participant dialogue, richness of narratives, recurrence of meaningful patterns across interviews, and coherence of emerging themes. Recruitment continued until sufficient information power was achieved, with additional interviews contributing limited new insights or substantially different interpretations, indicating that the dataset was adequate to address the study aim.

A total of 32 eligible patients were approached to participate in the study. Of these, 25 agreed to participate, while seven declined, primarily because of time constraints or lack of interest. Clinical staff assisted only in identifying potentially eligible patients and did not influence recruitment decisions. The final sample comprised 25 participants and reflected the intended maximum variation in age, gender, educational background, employment status, and duration of heart failure. A summary of participant characteristics is presented in Table 1.

### 2.3. Study Setting

The study was conducted within a large medical city in Riyadh, Saudi Arabia, comprising four general tertiary-care hospitals, a specialized cardiac center, and affiliated primary healthcare and outpatient services. The medical city serves a diverse population from Riyadh and surrounding regions and provides comprehensive cardiovascular services, including specialized HF outpatient clinics, advanced cardiac diagnostics, inpatient cardiac care, and long-term chronic disease management. Participants were recruited primarily from outpatient HF and cardiac follow-up clinics, where patients receive ongoing multidisciplinary management for chronic HF. These clinics provide routine medical follow-up, medication management, patient education, and long-term monitoring for individuals living with HF. The setting was considered appropriate for this study because it provides access to a heterogeneous population of patients with varying clinical presentations, socioeconomic backgrounds, and durations of illness experience. Interviews were conducted in private consultation rooms within the hospital to ensure participant comfort, confidentiality, and minimal interruption during data collection.

### 2.4. Ethical Considerations

Ethical approval for this study was obtained from the King Saud University Institutional Review Board (IRB) prior to the commencement of data collection (Approval No. E-25-9965; 1 January 2026). Administrative approval was also obtained from the participating clinical sites before participant recruitment. Eligible participants were approached in outpatient cardiac clinics, provided with detailed verbal and written information regarding the study aims and procedures, and invited to participate voluntarily. Written informed consent was obtained from all participants before participation, including consent for audio recording of the interviews. Participants were informed of their right to decline participation or withdraw from the study at any stage without affecting their care or treatment. To ensure confidentiality and data security, all identifying information was removed from interview transcripts, and participants were assigned anonymized identification codes (e.g., P1, P2). Audio recordings, transcripts, and all study-related materials were securely encrypted and stored on password-protected systems accessible only to the research team. Participants did not receive any financial compensation or reimbursement for their participation.

### 2.5. Data Collection

Participants were recruited purposively from outpatient HF and cardiac follow-up clinics with the assistance of clinical staff, who identified potentially eligible participants based on the study inclusion criteria. Clinical staff assisted only with identifying eligible participants and did not influence recruitment decisions. Eligible participants received verbal and written information about the study and were invited to participate voluntarily. Data were collected through semi-structured, in-depth interviews conducted between January and April 2026. Interviews were conducted face-to-face in private consultation rooms within the hospital, providing a quiet and confidential environment conducive to open and in-depth discussion.

The interviews were conducted individually by male researchers with doctoral-level training and experience in qualitative health research and interview-based studies. No prior relationship existed between the researchers and participants before recruitment. Interviews were conducted in Arabic or English according to participant preference, lasted approximately 40–60 min, and were audio-recorded with participants’ consent. No participants required or requested the presence of a family member during the interviews. Field notes were documented following each interview to capture contextual observations and preliminary reflections, and data collection and preliminary analysis occurred concurrently.

An interview guide informed by the study aims and relevant heart failure literature was used to facilitate discussion. Consistent with the interpretivist orientation of the study, interviews were conducted in a flexible and participant-centred manner, allowing participants to construct and express their own meanings and experiences. Rather than restricting data collection to predefined categories, the semi-structured format enabled the exploration of culturally embedded beliefs, emotional responses, and social contexts. No standardized HRQoL instrument was used, as the study sought to explore HRQoL as a subjective, culturally situated experience through open-ended, in-depth interviews. Accordingly, the interview guide included questions addressing the physical, emotional, social, and spiritual dimensions of living with heart failure (e.g., “Can you describe how heart failure has affected your daily life?” and “How has your condition influenced your relationships and emotional well-being?”). Within this study, HRQoL was understood as participants’ subjective perceptions of how heart failure influenced their overall well-being and everyday lives, encompassing physical functioning, emotional well-being, social participation, family roles, independence, spiritual well-being, and confidence in managing healthcare needs. This conceptualization informed the interview process and supported exploration of the personal and culturally situated meanings participants attributed to living with heart failure.

All Arabic interviews were transcribed verbatim in the original language to preserve participants’ expressions, contextual meanings, and culturally specific interpretations. Transcripts were reviewed against the audio recordings to ensure accuracy and completeness. Translation into English was undertaken after initial transcription and preliminary analysis by a bilingual researcher with expertise in qualitative methods. The translation process emphasized semantic and cultural equivalence rather than literal word-for-word translation, with particular attention to culturally embedded concepts related to spirituality, family relationships, coping, illness beliefs, and social roles [28]. A second bilingual researcher independently reviewed all participant quotations included in the manuscript against the original Arabic transcripts and audio recordings to verify conceptual accuracy and preserve participants’ intended meanings and cultural context. Any discrepancies or uncertainties were discussed among the research team until agreement was reached regarding the most appropriate interpretation. This iterative process helped preserve participants’ intended meanings and enhanced the interpretive fidelity of the findings.

### 2.6. Researcher Characteristics and Reflexivity

The interviews were conducted by male qualitative researchers with doctoral-level training in nursing and experience in qualitative health research. The research team consisted of Saudi academic researchers who were familiar with the cultural, social, and healthcare context surrounding chronic illness and heart failure in Saudi Arabia. As Saudi male researchers interviewing Saudi adults living with heart failure, the researchers occupied an insider cultural position while recognizing that their gender, professional identities, and clinical backgrounds could shape participant interactions and influence both data collection and interpretation. This cultural familiarity facilitated rapport with participants, supported culturally sensitive communication, and encouraged open discussion of personal, emotional, social, and spiritual experiences of living with heart failure. The researchers also recognized that their insider cultural position and professional identities could have influenced what participants chose to disclose, what they assumed was already understood, or what remained unspoken during the interviews. These possibilities were considered throughout reflexive discussions and memo writing during data collection and analysis.

Consistent with the study’s interpretivist epistemology and Braun and Clarke’s reflexive thematic analysis, the researchers acknowledged their active role in the co-construction of knowledge rather than assuming a neutral or objective position [29]. They recognized that their professional backgrounds in nursing and prior clinical and academic experiences could predispose them to interpret heart failure primarily through a clinical lens. To promote reflexivity, the principal researcher maintained reflexive memos throughout data collection and analysis to document methodological decisions, emerging interpretations, and potential assumptions. These reflections were regularly discussed within the research team during data collection, coding, and theme development to critically examine emerging interpretations, challenge implicit assumptions, and ensure that participants’ perspectives remained central to the analytic process.

Reflexive engagement was further supported through iterative review of interview transcripts, collaborative team discussions, and the ongoing critical examination of developing interpretations. These strategies enhanced transparency, analytical rigour, and interpretive depth, ensuring that the findings reflected rigorous and reflexive engagement with participants’ accounts rather than being presented as objective or neutral representations of their realities.

### 2.7. Data Analysis

Data were analyzed using Braun and Clarke’s reflexive thematic analysis (RTA) approach [23,24,25]. RTA was selected because of its theoretical flexibility and alignment with the study’s interpretivist epistemology, enabling exploration of patterns of shared meaning while acknowledging the contextual, subjective, and interpretive nature of knowledge construction. The final dataset comprised 25 verbatim interview transcripts from Saudi adults living with heart failure. Data analysis occurred concurrently with data collection, allowing emerging insights to inform ongoing engagement with the data and refinement of the analytic process. All interview transcripts were included in the final analysis.

Data analysis was initially undertaken using the original Arabic transcripts to preserve participants’ meanings, expressions, and culturally embedded interpretations. Familiarization, coding, and preliminary theme development were therefore conducted using the Arabic-language data. Translation into English was performed after initial coding and preliminary theme development to facilitate reporting and collaborative discussion among the research team. Particular attention was given to maintaining semantic accuracy and conceptual equivalence, especially for culturally significant concepts related to spirituality, family obligations, dependency, coping, and illness experiences. Culturally specific Arabic expressions (e.g., *Subhan Allah*, *Wallah*, *qadar*, *tawakkul*, and *sabr*) were retained in transliterated form where appropriate and accompanied by brief contextual explanations to preserve their cultural and interpretive meanings for an international readership. Bilingual researchers reviewed translated transcripts and all participant quotations against the original Arabic transcripts to ensure that participants’ intended meanings and cultural context were preserved.

The analysis followed the six recursive phases of Braun and Clarke’s reflexive thematic analysis: (1) familiarization with the data through repeated reading of transcripts; (2) generation of initial codes representing meaningful features of the dataset; (3) development of candidate themes through identification of patterns of shared meaning; (4) reviewing and refining themes in relation to coded extracts and the complete dataset; (5) defining and naming themes according to their central organizing concepts; and (6) producing the final analytic narrative. During familiarization, transcripts were read repeatedly to develop a comprehensive understanding of participants’ experiences and identify preliminary patterns of meaning. Initial coding was conducted inductively, focusing on both explicit content and underlying meanings within participants’ narratives related to living with heart failure and its influence on HRQoL. Coded segments were subsequently compared across the dataset to identify broader patterns of shared meaning and develop candidate themes. Throughout this process, the researchers moved iteratively between individual coded extracts, the broader dataset, and ongoing reflexive discussions to examine how conceptually related codes could be integrated into higher-order interpretive themes that captured patterned meanings across participants’ accounts while remaining grounded in the dataset as a whole. Themes were iteratively reviewed, refined, reorganized, and interpreted in relation to the complete dataset to ensure they captured coherent patterns of shared meaning rather than descriptive summaries of participants’ accounts. Consistent with Braun and Clarke’s reflexive thematic analysis, themes were understood as interpretive constructions developed through the researchers’ active and reflexive engagement with the data, rather than as objective categories emerging independently from participants’ accounts. Reflexive practices, including analytic memo writing, critical reflection, and research team discussions, were integrated throughout the analytic process to examine how researchers’ perspectives, professional backgrounds, and contextual knowledge informed the interpretation of the data.

NVivo 14 (Lumivero, Denver, CO, USA) was used to support qualitative data management, including the organization of transcripts, systematic coding, retrieval of coded extracts, and management of analytic materials. The software served as a tool to organize and manage the dataset; however, coding, theme development, and all analytic decisions remained the responsibility of the researchers and were guided by the principles of Braun and Clarke’s reflexive thematic analysis.

Member checking was not undertaken as a deliberate methodological decision consistent with the interpretivist orientation and principles of Braun and Clarke’s reflexive thematic analysis. Rather than seeking participant validation of interpretations, the research team prioritized sustained engagement with the data through repeated reading of transcripts, iterative coding, reflexive memo writing, and ongoing analytic discussions to support interpretive depth and meaning-making. Additional strategies used to enhance the rigour and trustworthiness of the study are described in the following section.

### 2.8. Rigor and Trustworthiness

**Figure 1:**
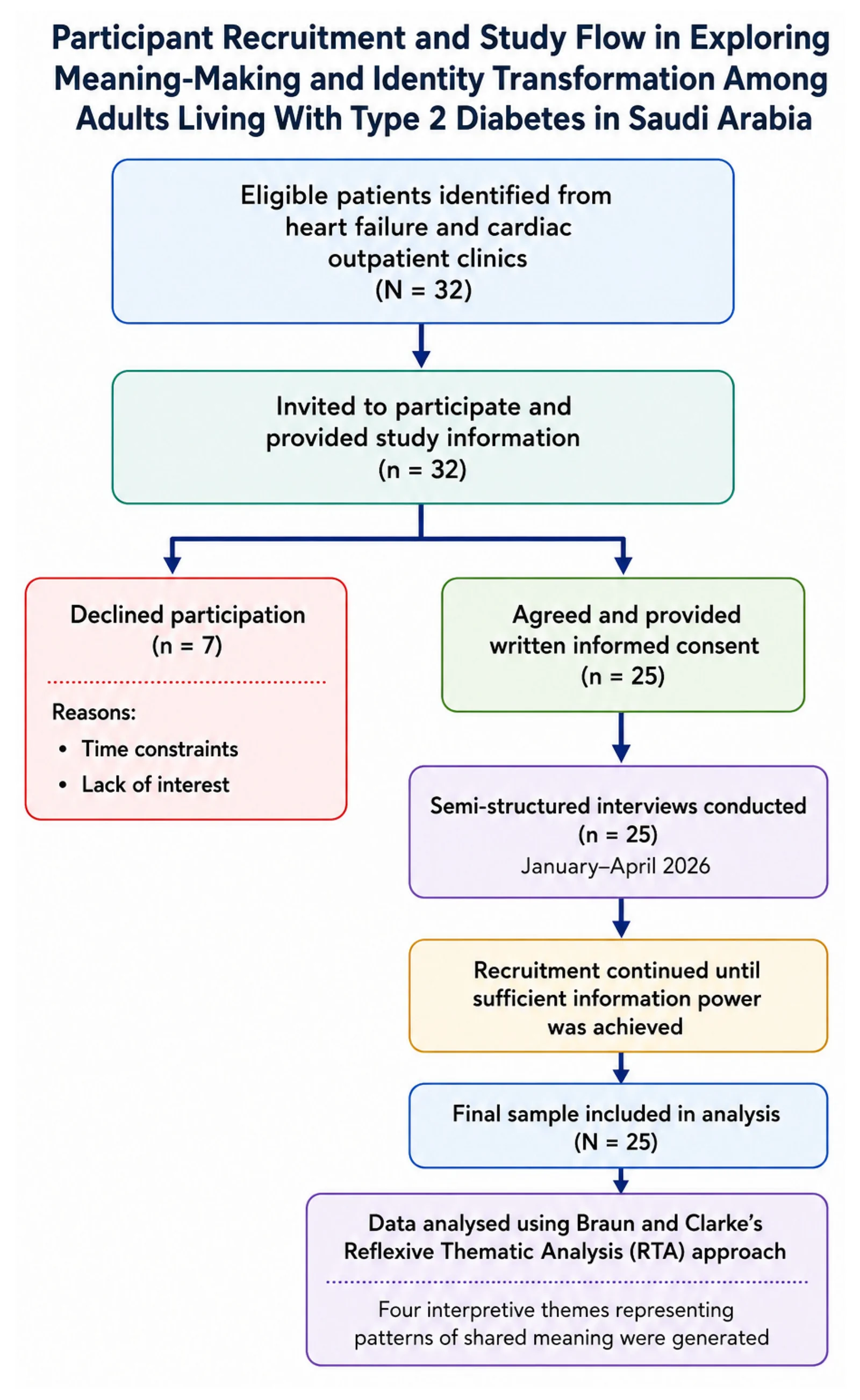
Participant recruitment, data collection, and reflexive thematic analysis workflow.

Consistent with the study’s interpretivist orientation and the principles of Braun and Clarke’s reflexive thematic analysis (RTA) [23,25,30], several strategies were employed to enhance the rigour and trustworthiness of the study. Drawing on the concepts of credibility, dependability, confirmability, and transferability described by Lincoln and Guba [30], interpretive credibility was strengthened through prolonged engagement with the data, iterative analysis, and ongoing reflexive engagement throughout data collection, coding, and theme development. Dependability and confirmability were supported through systematic documentation of methodological and analytic decisions, including reflexive memo writing and collaborative review of coding and theme development. Verbatim participant quotations were incorporated to enhance transparency and illustrate the interpretive basis of the findings. Transferability was supported through purposive maximum-variation sampling and rich description of the study context, participants, and findings, enabling readers to assess the applicability of the results to other contexts. In accordance with the Consolidated Criteria for Reporting Qualitative Research (COREQ), a participant flow diagram (Figure 1) is provided to summarize participant recruitment and enrolment.

## 3. Results

### 3.1. Characteristics of Participants

The sample comprised 25 participants with a mean age of 58.4 ± 12.6 years (range 34–79), of whom 64% were male. Educational attainment varied, with 16% having no formal education, 44% completing primary or secondary education, and 40% holding a university degree or higher. Most participants were unemployed or retired (68%), while 32% were employed. The mean duration of heart failure was 6.2 ± 4.1 years, with 56% living with the condition for five years or more. The majority were married (80%) and living with family members (88%), reflecting the strong familial context of the sample.

### 3.2. Themes Derived from the Analysis

Four interconnected themes were generated through the analysis: (1) The Physical Burden of HF on Daily Life, (2) Emotional Distress and the Psychological Weight of Dependency, (3) Disrupted Social Roles and Reliance on Family Support, and (4) Coping Through Faith, Cultural Values, and Trust in Healthcare. Supplementary Table S1 presents the themes, subthemes, and representative quotations.

The themes presented below represent interpretive patterns of shared meaning constructed through reflexive engagement with the data, rather than direct categorizations of participants’ accounts. While grounded in participants’ narratives, each theme captures a distinct dimension of how heart failure was experienced and understood. Subthemes are used to illustrate specific facets of these broader patterns, while maintaining clear conceptual boundaries between themes.

### 3.3. Physical Dimensions of Health-Related Quality of Life in Heart Failure

**Theme 1. The Physical Burden of Heart Failure on Daily Life**

Persistent physical symptoms and functional decline were central features of participants’ lived experiences with HF. These limitations shaped their day-to-day routines and contributed to emotional distress, loss of independence, and reduced social involvement.

#### 3.3.1. Constant Fatigue, Breathlessness, and Difficulty Moving

Persistent fatigue and breathlessness emerged as dominant features shaping participants’ daily experiences of HF. These symptoms disrupted mobility, reduced independence, and limited engagement in routine social and religious activities. One participant explained:

“*I get tired after just a few steps. I feel out of breath, and my body has no energy at all. Even small movements make me feel weak*.”.

(P7)

Several participants described how physical exhaustion interfered with routine activities, including prayer practices and personal care. One participant reflected:

“*My legs can’t hold me when I walk or stand. Even when I go make wudu, I get tired. My legs feel too weak to carry me because of the exhaustion*.”.

(P1)

Others emphasized dramatic changes in their ability to remain socially and physically active:

“*I used to go everywhere and stay active, but now I can barely go out or do my daily activities because I constantly feel exhausted*.”.

(P19)

For some participants, physical decline was also accompanied by spiritual reflection and awareness of aging and vulnerability:

“*Subhan Allah* (Glory be to God)*, no one stays the same. I used to walk easily, but now I get tired after ten minutes. Only the Face of Allah lasts forever*.”.

(P12)

#### 3.3.2. Sleep Disturbances Related to HF Symptoms

Sleep disruption emerged as a persistent challenge affecting participants’ physical recovery, emotional well-being, and daily functioning. Participants frequently described nocturnal dyspnea, orthopnea, coughing, and repeated awakenings that contributed to exhaustion and irritability during the day. One participant stated:

“*When I sleep, I feel short of breath. I use two or three pillows to feel better. My sleep is very interrupted…I wake up many times during the night*.”.

(P2)

Others described the cumulative effect of poor sleep on energy levels and emotional well-being:

“*I don’t feel energetic in the morning. I feel tired, weak, and exhausted because of lack of sleep. I’m often irritable and frustrated*.”.

(P21)

#### 3.3.3. Medication Side Effects Limiting Daily Life

Medication-related challenges added another layer of physical and psychological burden to participants’ daily lives. Participants described difficulties related to polypharmacy, medication side effects, and managing complex treatment regimens over prolonged periods. One participant shared:

“*I take six or seven pills a day. Some make me dizzy, my head spins. They told me they lower my blood pressure, and some give me headaches*.”.

(P8)

Others reflected on long-term complications associated with prolonged medication use:

“*I’ve been on medications for over 15 years. The doctor said they caused stomach ulcers and gave me another medicine to protect my stomach*.”.

(P13)

Beyond physical symptoms, participants’ accounts suggest that fatigue, breathlessness, sleep disruption, and medication-related challenges collectively altered how they experienced their bodies and engaged with everyday life. Rather than representing isolated physical impairments, these interconnected experiences undermined participants’ sense of bodily control, independence, and normality. Consequently, the physical burden of heart failure extended beyond functional limitation into an existential disruption of identity, shaping how participants understood themselves and their place within daily life.

### 3.4. Psychological Dimensions of Health-Related Quality of Life

**Theme 2. Emotional Distress and the Psychological Weight of Dependency**

Participants described living with HF as emotionally overwhelming, with persistent fear, uncertainty, and psychological distress shaping their daily lives. Emotional suffering was often intensified by fears of sudden deterioration, loss of independence, and increasing reliance on family members for support.

#### 3.4.1. Fear and Worry About Health

Fear of sudden deterioration and uncertainty about the future emerged as central emotional experiences among participants living with HF. Feelings of anxiety were particularly evident during episodes of shortness of breath, palpitations, or when participants were alone. One participant explained:

“*When I’m alone, I start feeling scared and anxious, like something could happen to me at any moment*.”.

(P3)

Several participants expressed intense fear during episodes of worsening symptoms:

“*Every time I feel my heart racing, I start fearing death. I shake and feel like I’m going to die*.”.

(P11)

Nocturnal symptoms were especially distressing and often intensified fears of death and suffocation:

“*When I sleep on my back, I get severe shortness of breath and feel like I am choking. I feel like I am going to die*.”.

(P14)

#### 3.4.2. Stress and Difficulty Coping

The ongoing demands of managing HF, including medication schedules, medical appointments, and unpredictable symptoms, contributed to emotional exhaustion and difficulty coping. Several participants described feeling overwhelmed by the constant physical and psychological burden of the illness. One participant stated:

“*When I get really fatigued and feel like my heart is about to jump out of my chest, I get crazy stressed and don’t know what to do*.”.

(P6)

Others expressed frustration with the continuous demands of treatment and follow-up care:

“*Taking medications on time, the appointments, the follow-ups… it all got exhausting. Wallah* (I swear by God)*, I’m just fed up*.”.

(P8)

#### 3.4.3. Feeling Like a Burden on Family

Feelings of dependency on family members created significant emotional distress for many participants. As physical limitations progressed, participants described increasing reliance on spouses, children, and relatives for transportation, daily care, and emotional support. One participant shared:

“*My kids are stressed over me. They check my pressure, sit by my side, carry all my worries*.”.

(P14)

Others expressed feelings of guilt and loss of independence associated with becoming dependent on family caregivers:

“*I feel like I’m a burden on my family. My son drives me everywhere, my wife does everything—even helps me drink. I don’t have the strength to take care of myself anymore*.”.

(P9)

These emotional responses extend beyond psychological reactions to illness, reflecting a deeper tension between dependency and identity within the sociocultural context. Fear of becoming a burden was closely linked to culturally embedded expectations of independence, productivity, and family roles. Consequently, emotional distress arose not only from the illness itself but from a perceived disruption of one’s moral and social standing within the family.

### 3.5. Social Dimensions of Health-Related Quality of Life

**Theme 3. Disrupted Social Roles and Reliance on Family Support**

HF substantially disrupted participants’ social participation, family responsibilities, and occupational roles, often altering their sense of identity, independence, and social relevance within family and community life.

#### 3.5.1. Reduced Social Activity and Isolation

Increasing physical limitations and fatigue led many participants to withdraw from social activities and reduce participation in gatherings they previously enjoyed. Participants described feeling socially restricted, isolated, and unable to maintain previous levels of community engagement. One participant stated:

“*Because of my illness, I can’t go out like before. I’m always stuck at home now*.”.

(P11)

Participants further described difficulty participating in social occasions because of exhaustion and reduced physical stamina:

“*When I go to social occasions, I can’t stay long. I get tired fast and leave early*.”.

(P4)

Some participants also perceived subtle social distancing from others following their illness:

“*I used to get invited to weddings and gatherings. But now my friends stopped inviting me like before—they know my health*.”.

(P21)

#### 3.5.2. Work and Daily Responsibilities Affected

HF substantially affected participants’ ability to maintain employment and fulfill daily responsibilities, often resulting in financial strain and loss of occupational identity. Participants described reduced physical capacity, inability to sustain previous work demands, and increased dependence on social or family support. One participant shared:

“*I used to work multiple jobs to provide for my kids. Now I can barely go out to earn anything*.”.

(P17)

Others described being forced to leave employment because of worsening health conditions:

“*I resigned, and now I live on social security because I can’t work anymore*.”.

(P2)

#### 3.5.3. Altered Role Within the Family

HF frequently altered family dynamics and caregiving roles, affecting participants’ sense of identity, dignity, and independence. Several participants described a transition from being caregivers and providers to becoming increasingly dependent on family members for daily support. One participant explained:

“*My role changed completely. I used to be the head of the house. Now they bring me food, feed me, take care of me*.”.

(P10)

Others described role reversals within the family structure as children assumed greater responsibility for household and caregiving duties:

“*My eldest son takes care of everything now… it’s like he became the man of the house*.”.

(P15)

The disruption of social roles illustrates how heart failure reshaped participants’ social identities, particularly within a cultural context that emphasizes family responsibility and contribution. Loss of independence was experienced not merely as functional decline, but as a redefinition of one’s role within the family. This transition from provider or caregiver to care recipient carried significant emotional and symbolic implications.

### 3.6. Spiritual and Cultural Influences on Health-Related Quality of Life

**Theme 4. Coping Through Faith, Cultural Values, and Trust in Healthcare**

Participants described multiple coping strategies that helped them manage the physical, emotional, and social burden of HF. Spiritual beliefs, family support, positive thinking, and trusting relationships with healthcare providers emerged as important sources of resilience and emotional stability.

#### 3.6.1. Faith and Spiritual Practices as Support

Faith and spiritual practices emerged as central coping mechanisms that provided participants with comfort, acceptance, and emotional reassurance while living with HF. Participants frequently described relying on Islamic beliefs and spiritual practices to cope with uncertainty, fear, and physical suffering. One participant stated:

“*Islam makes us trust God. Death, fate, sickness—they’re all in His hands*.”.

(P9)

Several participants expressed how religious practices provided emotional relief and strengthened their ability to cope during periods of illness:

“*When I feel very sick, I read the Quran. The verses comfort me mentally. I say, ‘Oh God, I put my trust in You*.”.

(P3)

#### 3.6.2. Maintaining a Positive Mindset

Several participants described actively maintaining hope and a positive outlook despite the ongoing challenges of living with HF. Participants emphasized the importance of continuing daily activities, avoiding excessive focus on illness, and sustaining emotional resilience. One participant explained:

“*I try to forget about my illness because if I keep thinking about it, I’ll go crazy. I try to go out, walk around, live my life*.”.

(P2)

Others expressed determination to remain active and engaged despite physical limitations:

“*A person shouldn’t give up to illness. You keep working and moving till your last day*.”.

(P21)

#### 3.6.3. Support from Family, Friends, and Relatives

Support from family members, relatives, and close friends was described as an important source of emotional comfort and psychological relief. Participants emphasized the value of social connection, companionship, and emotional reassurance in helping them cope with the challenges of HF. One participant stated:

“*When I sit with my friends, especially my childhood buddies, I forget my illness. I relax a bit*.”.

(P19)

Others described the emotional comfort gained through family interactions and social gatherings:

“*When I meet my brothers, sisters, cousins… the conversations ease my mind*.”.

(P4)

Several participants also highlighted the importance of close family support during periods of illness and vulnerability:

“*Having my married sons around me makes me feel emotionally supported. They’re really caring*.”.

(P5)

#### 3.6.4. Communication and Understanding of Treatment

Clear and respectful communication with healthcare providers contributed positively to participants’ confidence, understanding, and treatment adherence. Participants valued when doctors and nurses explained medical information in understandable ways and responded attentively to their concerns. One participant explained:

“*My doctor listens to me—really listens—and that makes me feel safe about my treatment*.”.

(P2)

Others described feeling more comfortable engaging in their care when healthcare providers communicated clearly:

“*I feel free to ask any question because they explain everything clearly*.”.

(P18)

Several participants emphasized the importance of simplified explanations in helping them understand medications and treatment plans:

“*Medical words confuse me, but the nurse explains them in a way I get. That helps me not freak out*.”.

(P10)

Participants also noted that understanding the purpose of medications improved adherence and confidence in treatment:

“*When I understand why I take each pill, it’s easier to stick to them*.”.

(P3)

#### 3.6.5. Trust in Providers and Access to Care

Trust in healthcare providers influenced participants’ sense of safety, reassurance, and confidence in managing HF. Participants described feeling more secure when healthcare professionals demonstrated competence, respect, and genuine concern for their well-being. One participant stated:

“*I trust the doctors—they actually try to help me. That makes me feel in good hands*.”.

(P21)

Others emphasized the importance of respectful treatment and supportive interactions with healthcare staff:

“*The staff treats me with respect, and that makes a big difference*.”.

(P7)

Several participants described feeling reassured when following medical advice and treatment recommendations:

“*I feel safe following their instructions… I know they’ve got me covered*.”.

(P5)

Despite these positive experiences, some participants reported frustration related to delayed appointments and difficulty accessing timely care:

“*Appointments take too long to schedule, and that worries me*.”.

(P10)

For some participants, the emergency department became an alternative source of reassurance during periods of symptom worsening:

“*When I feel worse, I go to the ER because it’s faster. It gives me peace of mind*.”.

(P11)

Faith, family relationships, and trust in healthcare functioned not merely as coping resources but as interconnected interpretive frameworks through which participants made sense of their illness. The invocation of religious beliefs reframed heart failure as part of a divinely ordained experience, enabling acceptance and psychological adjustment. This suggests that coping was not merely an individual strategy but a culturally embedded process that integrated spirituality, family support, and trust in healthcare into a coherent system of meaning-making.

Overall, participants’ experiences illustrate that health-related quality of life was shaped by the dynamic interaction between physical limitations, emotional distress, disrupted social roles, and culturally embedded coping processes. Rather than operating independently, these dimensions influenced one another, highlighting HRQoL as a multidimensional and culturally situated experience of living with heart failure.

## 4. Discussion

This study provides an in-depth exploration of how a group of Saudi adults receiving outpatient care at a single tertiary medical city experience the physical, emotional, social, and spiritual consequences of HF within a culturally embedded context. The findings suggest that HF extends beyond physical symptom burden and may substantially affect identity, independence, social participation, emotional well-being, and religious practice. Across the four interconnected themes, participants described HF as a disruptive experience that altered daily functioning, family roles, psychological stability, and coping processes. Although many of these experiences are consistent with the previous HF literature, the findings also highlight culturally specific dimensions shaped by Islamic beliefs, collectivist family structures, and socially embedded understandings of caregiving, dependency, and resilience. These interpretations should, however, be considered in light of the study’s context and sample, and they are not intended to represent all Saudi adults living with HF.

### 4.1. The Physical Burden of Heart Failure on Daily Life

Consistent with previous HF research, participants described fatigue and breathlessness as persistent and disabling symptoms that negatively affected quality of life and functional capacity [31]. However, the present findings further suggest that, within this sample, physical limitations also disrupted religious practices, family participation, and social connectedness.

Participants’ descriptions of difficulty performing activities such as ablution, prayer, family visits, and social gatherings highlight the broader social and spiritual consequences of HF within Saudi society. Similar findings have been reported among individuals living with HF and other cardiovascular conditions in Arab and Middle Eastern settings, where family relationships, social participation, and religious practices influence illness experiences [20,32].

Participants also described substantial challenges related to polypharmacy, including dizziness, hypotension, gastrointestinal discomfort, and difficulty managing complex medication regimens. These experiences contributed to treatment burden and reduced participants’ confidence in managing their condition independently. The previous HF literature has similarly identified polypharmacy as an important contributor to reduced quality of life, treatment fatigue, and poor adherence [33,34].

Overall, the findings suggest that the physical burden of HF extends beyond symptom experience and may influence autonomy, daily functioning, religious participation, and social engagement. Although many physical symptoms described by participants are consistent with the international HF literature, the findings also highlight culturally specific consequences related to spirituality, family interaction, and participation in socially meaningful activities within the Saudi context. These culturally specific interpretations should be understood as context-bound and may vary across different regions and healthcare settings within Saudi Arabia.

### 4.2. Emotional Distress, Fear, and the Psychological Impact of Dependency

Participants described HF as emotionally overwhelming, with persistent fear, uncertainty, and psychological distress shaping their daily experiences. Fear was particularly associated with sudden symptom deterioration, nocturnal breathlessness, palpitations, and uncertainty regarding disease progression and death. These findings are consistent with the previous HF literature, which identifies uncertainty and fear as central emotional dimensions of living with chronic cardiac illness [35,36].

Participants also described significant emotional distress associated with increasing dependency on family members for transportation, personal care, and daily activities. Within the Saudi sociocultural context, where independence, productivity, and fulfillment of family responsibilities are highly valued, this transition from caregiver and provider to care recipient was experienced as emotionally difficult and identity-threatening. Feelings of shame, guilt, and reduced self-worth were particularly evident among male participants who perceived loss of employment and diminished ability to provide for their families as a disruption to their social and familial roles.

This pattern reflects the gendered nature of family responsibility within Saudi society, where men are traditionally positioned as primary providers and heads of household. For several male participants, the inability to sustain this role appeared to intensify feelings of dependency beyond the physical limitations of HF itself, extending into a perceived loss of masculine identity and social standing. Female participants in this study did not describe comparable disruption to a provider identity; however, as the sample was predominantly male and gender was not a specific analytic focus of this study, these patterns should be interpreted as illustrative rather than as evidence of a systematic gender difference in emotional response to HF.

These findings reflect the concept of biographical disruption, whereby chronic illness interrupts previously valued identities, social roles, and assumptions about independence and future life trajectories. Similar experiences of role loss, dependency, and altered self-identity have been reported among individuals living with chronic illness and HF across diverse cultural settings [37,38].

Participants further described emotional exhaustion associated with continuous symptom monitoring, medication management, medical appointments, sleep disruption, and uncertainty regarding disease progression. The cumulative burden of ongoing self-management contributed to stress, frustration, and psychological fatigue. The previous literature similarly suggests that the ongoing burden of symptom management, self-care, and treatment adherence in HF may contribute to anxiety, psychological distress, and reduced quality of life [39,40].

Overall, the findings indicate that the emotional burden of HF extends beyond fear of physical deterioration and encompasses experiences of dependency, role disruption, uncertainty, and psychological vulnerability. Although emotional distress is widely recognized within the international HF literature, the findings suggest that the emotional consequences of dependency and role loss may be intensified for some participants within this sample, particularly within family structures and culturally embedded expectations regarding independence, caregiving, and social responsibility.

### 4.3. Social Role Disruption and Family Dependence

Participants described HF as substantially restricting their ability to engage in social, familial, and community activities. Social withdrawal was often driven by fatigue, physical limitations, concerns about symptom exacerbation, and reduced confidence in participating in gatherings. Consistent with previous HF research, social isolation emerged as a common consequence of chronic illness and declining functional capacity [6,41]. However, within the Saudi sociocultural context, where social participation, hospitality, and family engagement are highly valued, these limitations represented more than reduced activity; they signified a loss of social connection, belonging, and social relevance [5].

Loss of employment and reduced work capacity emerged as important consequences of HF. Participants described being unable to sustain previous occupational responsibilities because of fatigue, recurrent symptoms, and declining physical function. Similar findings have been reported internationally, where HF is associated with reduced productivity, unemployment, and financial strain [5,6]. Beyond economic consequences, however, loss of employment also represented a disruption to personal identity, independence, and perceptions of self-worth, particularly among participants who viewed their provider role as central to family life.

Participants also described substantial changes in family dynamics as children and other relatives assumed increasing caregiving responsibilities. Although participants expressed gratitude for the support they received, these role reversals were often accompanied by feelings of guilt, dependency, and reduced autonomy. Within collectivist family structures, caregiving is frequently viewed as a shared responsibility; however, participants continued to experience emotional discomfort associated with becoming recipients rather than providers of care. Similar tensions between gratitude and dependency have been reported in studies of chronic illness within Arab and Gulf populations [5].

Gendered role shifts were evident among several participants, with some participants describing reliance on a spouse or child who “became the man of the house” (P15), reflecting disruption of traditional household authority. For men, this transition signified a loss of status beyond functional decline. Comparable experiences among women were not clearly captured, likely due to the predominantly male sample, highlighting a need for further gender-focused research.

Overall, the findings suggest that HF can substantially reshape social identity, occupational participation, and family relationships for the participants included in this study. Although social role disruption is widely reported among individuals living with HF, the findings indicate that its emotional impact may be amplified in this context, where family responsibility, social participation, and interdependence remain central components of personal identity and social belonging.

### 4.4. Coping Through Faith, Cultural Values, and Trust in Healthcare

Faith emerged as a prominent coping resource that enabled participants to make sense of their illness and manage the uncertainty associated with HF. Participants frequently described drawing upon Islamic beliefs, including qadar (divine decree), tawakkul (trust in God), and sabr (patience), to maintain hope, acceptance, and emotional stability. These findings are consistent with previous research demonstrating that religious and spiritual beliefs can strengthen resilience, reduce psychological distress, and facilitate adaptation to chronic illness among Muslim populations [42,43].

Participants also described actively maintaining a positive outlook despite ongoing physical limitations and uncertainty. Strategies such as focusing on daily achievements, remaining socially engaged, sustaining routine activities, and reframing illness experiences helped participants preserve a sense of purpose and control. Similar coping processes have been described in previous HF research, where positive cognitive reframing has been associated with improved psychological adjustment and resilience [44].

Family support emerged as a central coping resource, providing practical assistance, emotional reassurance, and companionship. Reflecting the collectivist nature of Saudi society, coping was often experienced as a shared family process involving collective caregiving and decision-making, extending beyond the immediate household [45,46]. Participants also highlighted the importance of clear, respectful communication with healthcare professionals. Effective communication enhanced understanding, confidence, and adherence, whereas delays and limited explanations contributed to frustration and uncertainty. These findings underscore the importance of patient-centered communication, continuity of care, and culturally responsive education in supporting long-term HF self-management [47]. In practice, these findings suggest potential value in culturally sensitive communication strategies, including clear explanations, family-inclusive discussions, and attention to patients’ religious and social contexts within HF management.

Overall, coping with HF reflects a dynamic interplay between spirituality, family support, personal resilience, and healthcare experiences, with Islamic beliefs and collective caregiving representing key contextual influences. Overall, HF is experienced not merely as a biomedical condition but as a social, emotional, and spiritual phenomenon shaped by cultural norms, underscoring the need for holistic, culturally responsive care. However, the extent to which these findings reflect broader populations should be interpreted cautiously given the study setting.

## 5. Implications

The findings suggest several implications for clinical practice and service delivery. Healthcare providers, particularly nurses, may benefit from adopting holistic, culturally sensitive approaches that address the physical, psychological, social, and spiritual dimensions of health-related quality of life. Routine assessment of these domains may help identify unmet needs and tailor care, particularly in relation to role disruption, dependency, and the impact of illness on religious practices. Cardiac clinics may consider strengthening structured patient education and continuity of care to support self-management and long-term adaptation. Integrating regular assessment of health-related quality of life into follow-up care may further support patient-centered care. Given the central role of family in the Saudi context, family involvement and education may be beneficial in supporting caregiving, improving adherence, and facilitating shared decision-making. Similarly, acknowledging patients’ religious beliefs and supporting faith-based coping may enhance emotional well-being and adjustment to illness. Developing clear referral pathways for psychosocial support, including counseling and mental health services, may also help address the emotional burden associated with heart failure. Future research should extend these findings across diverse regions and populations to further explore their transferability and contextual relevance.

## 6. Strengths and Limitations

This study has several strengths, including addressing an important gap in qualitative heart failure research by exploring the experiences of Saudi adults within an underrepresented sociocultural context. The use of an interpretivist qualitative descriptive design and Braun and Clarke’s reflexive thematic analysis provided a rigorous and theoretically coherent approach for generating in-depth insights into the physical, emotional, social, and spiritual dimensions of living with HF. Semi-structured interviews generated rich, contextually meaningful accounts, while purposive maximum-variation sampling enhanced the diversity and transferability of perspectives. The study also demonstrated strong reflexive practices through attention to researcher positionality, memo writing, and team discussions, with Arabic-based analysis and bilingual review supporting the preservation of culturally embedded meanings. Collectively, the findings provide clinically relevant insights that emphasize the importance of holistic, culturally responsive HF care addressing symptom burden, psychological well-being, family involvement, spirituality, and patient–provider communication.

This study has several limitations that should be considered when interpreting the findings. Data were collected from participants receiving care at a single tertiary medical city, which may limit the transferability of findings to other regions, healthcare settings, or rural populations within Saudi Arabia. Additionally, participants were recruited from an outpatient setting and therefore may not reflect the experiences of individuals with more advanced HF or those requiring inpatient care. The sample size and qualitative design aim to provide depth of understanding rather than generalizability. Cultural interpretations identified in this study should therefore be understood as context-specific rather than representative of all Saudi adults living with HF. A further limitation of this study is that patients with more severe illness or those with limited access to outpatient care may be underrepresented. As participants were primarily recruited from outpatient settings, individuals with advanced heart failure, greater functional impairment, or barriers to healthcare access may not have been fully captured. This may limit the diversity of experiences represented and affect the transferability of the findings to more clinically complex or underserved populations.

## 7. Conclusions

This study provides important insight into the experiences of Saudi adults living with heart failure and suggests that living with the condition extends beyond physical symptoms to influence emotional well-being, social roles, family relationships, and spiritual life. Participants described heart failure as a disruptive illness that influenced multiple aspects of daily living and health-related quality of life. Although many experiences were consistent with the international heart failure literature, the findings also highlighted culturally embedded dimensions shaped by Islamic beliefs, family interconnectedness, and social expectations. The findings suggest the importance of adopting holistic and culturally responsive approaches to heart failure care that address psychological, social, spiritual, and family-related needs alongside physical symptom management. Understanding how patients interpret and cope with heart failure within their cultural and social environments may help healthcare professionals provide more person-centered and meaningful care. Taken together, these findings provide contextually situated insight into the experiences of participants in this study and may inform culturally responsive heart failure care in Saudi Arabia and similar cultural settings, while recognising that further research across diverse populations is needed to extend these insights.

## Figures and Tables

**Table 1:** Participant Characteristics (*N* = 25).

Characteristic	Category	*n* (%)/Mean ± SD
Age (years)	Mean ± SD	58.4 ± 12.6
	Range	34–79
Sex	Male	16 (64%)
	Female	9 (36%)
Education Level	No formal education	4 (16%)
	Primary/Secondary	11 (44%)
	University or higher	10 (40%)
Employment Status	Employed	8 (32%)
	Unemployed/Retired	17 (68%)
Duration of HF (years)	Mean ± SD	6.2 ± 4.1
	<5 years	11 (44%)
	≥5 years	14 (56%)
Marital Status	Married	20 (80%)
	Single/Widowed	5 (20%)
Living Arrangement	With family	22 (88%)
	Alone	3 (12%)

## Data Availability

The qualitative data generated during the current study are not publicly available because they contain potentially identifiable participant information but are available from the corresponding author upon reasonable request and with appropriate ethical approval.

## Supplementary Materials

The following supporting information can be downloaded at: https://www.mdpi.com/article/10.3390/healthcare14162458/s1, Table S1: Themes, Sub-Themes, and Participant Quotes.
