# Supplementary material for: Experiences of Saudi Adults Living with Heart Failure: A Reflexive Thematic Analysis of Health-Related Quality of Life
Source: Healthcare (Basel). 2026 Aug 9;14(16):2458. doi: 10.3390/healthcare14162458 (PMC13512441; doi:10.3390/healthcare14162458)
Supplement: Supplementary file 1 [file healthcare-14-02458-s001.zip › healthcare-4403325-supplementary.pdf]

Experiences of Saudi Adults Living with Heart Failure: A Reflexive Thematic Analysis of Health-Related Quality of Life

Table S1: Themes, Sub-Themes, and Participant Quotes

**Theme**	**Sub-Theme**	**Representative Participant Quotes**
**Theme 1: The Physical Burden of Heart Failure on Daily Life**	1.1 Constant fatigue, breathlessness, and difficulty moving	"I get tired after just a few steps. I feel out of breath, and my body has no energy at all. Even small movements make me feel weak." – P7, "My legs can't hold me when I walk or stand. Even when I go make wudu, I get tired." – P1, "I used to go everywhere… because I constantly feel exhausted." – P19, "SubhanAllah… I used to walk easily, but now I get tired after ten minutes." – P12.
	1.2 Sleep disturbances related to HF symptoms	"When I sleep, I feel short of breath. I use two or three pillows… I wake up many times during the night." – P2, "I don't feel energetic in the morning. I feel tired, weak, and exhausted because of lack of sleep." – P21.
	1.3 Medication side effects limiting daily life	"I take six or seven pills a day. Some make me dizzy, my head spins." – P8, "I've been on medications for over 15 years. The doctor said they caused stomach ulcers." – P13.
**Theme 2: Emotional Distress and the Psychological Weight of Dependency**	2.1 Fear and worry about health	"When I'm alone, I start feeling scared and anxious, like something could happen to me at any moment." – P3 "Every time I feel my heart racing, I start fearing death. I shake and feel like I'm going to die." – P11 "When I sleep on my back, I get severe shortness of breath and feel like I'm choking." – P14.
	2.2 Stress and difficulty coping	"When I get really fatigued… I get crazy stressed and don't know what to do." – P6, "Taking medications on time, the appointments… it all got

		exhausting. Wallah I'm just fed up." – P8.
	2.3 Feeling like a burden on family	"My kids are stressed over me. They check my pressure, sit by my side, carry all my worries." – P14, "I feel like I'm a burden on my family. My son drives me everywhere, my wife does everything—even helps me drink." – P9.
**Theme 3: Disrupted Social Roles and Reliance on Family Support**	3.1 Reduced social activity and isolation	"Because of my illness, I can't go out like before. I'm always stuck at home now." – P11, "When I go to social occasions, I can't stay long. I get tired fast and leave early." – P4, "I used to get invited to weddings… now my friends stopped inviting me like before." – P21.
	3.2 Work and daily responsibilities affected	"I used to work multiple jobs to provide for my kids. Now I can barely go out to earn anything." – P17, "I resigned, and now I live on social security because I can't work anymore." – P2.
	3.3 Altered role within the family	"My role changed completely. I used to be the head of the house. Now they bring me food, feed me, take care of me." – P10, "My eldest son takes care of everything now… it's like he became the man of the house." – P15.
**Theme 4: Coping Through Faith, Cultural Values, and Trust in Healthcare**	4.1 Faith and spiritual practices	"Islam makes us trust God. Death, fate, sickness—they're all in His hands." – P9, "When I feel very sick, I read the Quran. The verses comfort me mentally." – P3.
	4.2 Maintaining a positive mindset	"I try to forget about my illness… I try to go out, walk around, live my life." – P2, "A person shouldn't give up to illness. You keep working and moving till your last day." – P21.
	4.3 Support from family, friends, and relatives	"When I sit with my friends, especially my childhood buddies, I forget my illness. I relax a bit." – P19, "When I meet my brothers, sisters, cousins… the conversations ease my mind." – P4, "Having my married sons around me makes me

		feel emotionally supported. They're really caring." – P5.
	4.4 Communication and understanding of treatment	"My doctor listens to me—really listens—and that makes me feel safe about my treatment." – P2, "I feel free to ask any question because they explain everything clearly." – P18, "Medical words confuse me, but the nurse explains them in a way I get. That helps me not freak out." – P10, "When I understand why I take each pill, it's easier to stick to them." – P3.
	4.5 Trust in providers and access to care	"I trust the doctors—they actually try to help me. That makes me feel in good hands." – P21, "The staff treats me with respect, and that makes a big difference." – P7, "I feel safe following their instructions… I know they've got me covered." – P5, "Appointments take too long to schedule, and that worries me." – P10, "When I feel worse, I go to the ER because it's faster. It gives me peace of mind." – P11.
